# Robotic stereotactic body radiotherapy for localized prostate cancer: final analysis of the German HYPOSTAT trial

**DOI:** 10.1007/s00066-023-02044-2

**Published:** 2023-02-09

**Authors:** David Krug, Detlef Imhoff, Alfred Haidenberger, Nicole Heßler, Jane Schäfer, Stefan Huttenlocher, Georgios Chatzikonstantinou, Christoph Fürweger, Ulla Ramm, Inke R. König, Felix Chun, Michael Staehler, Claus Rödel, Alexander Muacevic, Reinhard Vonthein, Jürgen Dunst, Oliver Blanck

**Affiliations:** 1 Klinik für Strahlentherapie, Universitätsklinikum Schleswig-Holstein – Campus Kiel, Arnold-Heller-Str. 3, Haus L, 24105 Kiel, Germany; 2 Saphir Radiochirurgie Zentrum Frankfurt am Main und Norddeutschland, Kiel, Germany; 3 Klinik für Strahlentherapie, Universitätsklinikum Frankfurt, Frankfurt am Main, Germany; 4 Europäisches Radiochirurgie Centrum, Munich, Germany; 5 Institut für Medizinische Biometrie und Statistik, Universitätsklinikum Schleswig-Holstein, Lübeck, Germany; 6 Zentrum für Klinische Studien, Universität zu Lübeck, Lübeck, Germany; 7 German Center for Cardiovascular Research (DZHK), Lübeck, Germany; 8 Klinik für Urologie, Universitätsklinikum Frankfurt, Frankfurt am Main, Germany; 9 Urologische Klinik und Poliklinik, LMU Klinikum der Universität München, Munich, Germany

**Keywords:** CyberKnife, Hypofractionation, Radiation Oncology, Biochemical recurrence, Toxicity, Quality of life

## Abstract

**Purpose:**

We report results of the first German prospective multicenter single-arm phase II trial (ARO 2013-06; NCT02635256) of hypofractionated robotic stereotactic body radiotherapy (SBRT) for patients with localized prostate cancer (HYPOSTAT).

**Methods:**

Patients eligible for the HYPOSTAT study had localized prostate cancer (cT1‑3 cN0 cM0), Gleason score ≤ 7, prostate-specific antigen (PSA) ≤ 15 ng/ml, prostate volume ≤ 80 cm^3^, and an International Prostate Symptom Score (IPSS) ≤ 12. Initially, inclusion was limited to patients ≥ 75 years or patients 70–74 years with additional risk factors. The trial protocol was later amended to allow for enrolment of patients aged ≥ 60 years. The treatment consisted of 35 Gy delivered in 5 fractions to the prostate and for intermediate- or high-risk patients, also to the proximal seminal vesicles using the CyberKnife system (Accuray Inc., Sunnyvale, CA, USA). Primary endpoint was the rate of treatment-related gastrointestinal or genitourinary grade ≥ 2 toxicity based on the RTOG scale 12–15 months after treatment. Secondary endpoints were acute toxicity, late toxicity, urinary function, quality of life, and PSA response.

**Results:**

From July 2016 through December 2018, 85 eligible patients were enrolled and received treatment, of whom 83 could be evaluated regarding the primary endpoint. Patients mostly had intermediate-risk disease with a median PSA value of 7.97 ng/ml and Gleason score of 7a and 7b in 43.5% and 25.9% of patients, respectively. At the final follow-up 12–15 months after treatment, no patient suffered from treatment-related gastrointestinal or genitourinary grade ≥ 2 toxicity. Acute toxicity was mostly mild, with three grade 3 events, and the cumulative rate of grade ≥ 2 genitourinary toxicity was 8.4% (95% CI 4.1–16.4%). There were no major changes in urinary function or quality of life. The median PSA value dropped to 1.18 ng/ml 12–15 months after treatment. There was one patient who developed distant metastases.

**Conclusion:**

Robotic SBRT with 35 Gy in 5 fractions was associated with a favorable short-term toxicity profile. Recruitment for the HYPOSTAT‑2 trial (ARO-2018‑4; NCT03795337), which further analyses the late toxicity of this regimen with a planned sample size of 500 patients, is ongoing.

**Supplementary Information:**

The online version of this article (10.1007/s00066-023-02044-2) contains supplementary material, which is available to authorized users.

## Introduction

Radiation therapy is a standard treatment modality for patients with localized prostate cancer. The randomized controlled ProtecT trial showed equivalent overall survival for radiotherapy with short-term androgen deprivation therapy (ADT) compared to surgery for patients with localized prostate cancer [1]. Traditionally, radiotherapy has been applied using conventional fractionation with single fractions of 1.8–2 Gy in 5 fractions per week over the course of 7–8 weeks. However, preclinical and clinical studies have suggested an α/β value of 1.5–2 for prostate cancer [2], which is considerably lower than for many other tumors and even lower than the α/β value of surrounding organs such as the bladder and the rectum.

This provides a strong rationale for the use of hypofractionation in the treatment of prostate cancer. A multitude of randomized controlled trials have been conducted using moderate hypofractionation with single doses of 2.5–3.5 Gy [3]. Overall, moderate hypofractionation resulted in equivalent oncological outcomes. There were some signs of increased acute gastrointestinal (GI) and genitourinary (GU) toxicity, but chronic toxicity was similar. Moderate hypofractionation has been accepted as a standard of care for localized prostate cancer in almost all clinical situations [4].

Stereotactic body radiotherapy (SBRT) has been employed to treat extracranial tumors in bone, liver, and lung for over 20 years [5, 6]. Initial reports of SBRT for prostate cancer using ultra-hypofractionation with 5 fractions were published in 2009 [7]. In 2016, the German S3 guideline introduced SBRT for prostate cancer for the first time. The statement mandated the conduct of SBRT within the context of prospective clinical trials. Thus, the HYPOSTAT trial was designed [8]. We herein report the final results of the HYPOSTAT trial.

## Materials and methods

HYPOSTAT was a multicenter prospective single-arm trial (ARO 2013-06; NCT02635256) investigating the use of robotic SBRT using the CyberKnife-System (Accuray Inc., Sunnyvale, California, USA) for patients with localized prostate cancer. The study protocol has been published previously [8]. The study was approved by the ethics committee of the University of Lübeck (leading ethics committee, file number 13–052) as well as the local ethics committees at the participating sites.

### Eligibility criteria

The inclusion criteria were localized prostate cancer (i.e., no evidence of nodal or distant metastases), Gleason score ≤ 7, prostate-specific antigen (PSA) < 15 ng/ml, prostate volume < 80 cm^3^, IPSS ≤ 12, age > 75 years or age 70–75 years, and either PSA > 10 ng/ml and/or Gleason score 7b and/or Gleason score 7a with > 33% positive biopsy cores and/or cT > 2a and/or prostate volume > 60 cm^3^. Patients were not eligible in case of previous radiotherapy to the pelvis, contraindications against the implantation of fiducials, immunosuppressive therapy, relevant comorbidities interfering with the study procedures, or patient’s inability to understand or comply with the procedures.

At the time of study conception, the PREFERE trial, a large randomized controlled phase III trial comparing active surveillance, surgery, and different radiotherapy modalities, was recruiting in Germany [9]. If patients were eligible for the PREFERE-trial, enrolment into the PREFERE trial was favored. After the closure of the PREFERE trial in 2016, there was an amendment for the HYPOSTAT trial, allowing enrolment for patients aged ≥ 60 years without any additional age-based restrictions.

### Treatment planning and administration

Patients received implantation of 3–4 fiducials into the prostate. The planning CT scan (slice thickness ≤ 1.5 mm) was acquired ≥ 5 days after fiducial implantation. A minimum bladder filling of 20–30 ml was intended, placement of a foley catheter for potentially better urethra delineation was optional, as previously described [8]. To achieve optimal bowel preparation, self-administration of a daily rectal enema was intended. A planning MRI for precise prostate and critical structure delineation was required and coregistered with the planning CT scan as per the recommendations for SBRT practice [5, 6]. The gross tumor volume (GTV) was defined as the prostate for patients with low-risk prostate cancer. For intermediate- and high-risk patients, the proximal seminal vesicles (intersection between the seminal vesicles and a 1-cm expansion of the prostate contour) were included in the GTV.

The clinical target volume (CTV) was generated by expanding the GTV by 1–2 mm. A 3-mm margin was added around the CTV (dorsally 1 mm) to generate the planning target volume (PTV). The prescription dose was 35 Gy in 5 fractions (PTV V_35Gy_ ≥ 95%) to the PTV-encompassing 75–85% isodose (80–85% if the urethra was not contoured). The GTV should be covered by the 37.5 Gy or 38.5 Gy isodose for patients with low- (GTV V_37.5_ _Gy_ ≥ 95%) or intermediate- to high-risk prostate cancer (GTV V_38.5_ _Gy_ ≥ 95%), respectively. Organ at risk constraints and the urethral high-dose-sparing protocol have been published previously [8].

Treatment was performed using the CyberKnife system with translational and rotational tracking using the fiducial markers. Treatment was administered every other day until the post-PREFERE amendment and could be administered daily or every other day based on treatment center preference thereafter, with a maximum treatment duration of 2 weeks. As this amendment was designed, the decision was made to include a subgroup analysis according to the treatment schedule after discussion with the lead trial statistician (RV).

The use of ADT was allowed and discussed on an individual basis.

### Endpoints and statistical hypothesis

The primary endpoint was the rate of treatment-related grade ≥ 2 genitourinary (GU) and gastrointestinal (GI) toxicity based on the Radiation Therapy Oncology Group (RTOG) scale 12–15 months after treatment. Secondary endpoints were acute toxicity using the Common Terminology Criteria for Adverse Events (CTCAE) scoring system (version 4.03), late toxicity using the RTOG scoring system, urinary function based on the International Prostate Symptom Score (IPSS), quality of life (QOL) using the European Organisation for Research and Treatment of Cancer (EORTC) QOL questionnaire C30 and the Patient Oriented Prostate Utility Scale (PORPUS) questionnaires, as well as PSA response after radiotherapy.

Study visits were scheduled at each treatment session (V02–06), and 4–6 weeks (FU-01), 3 months (FU-02), 6–9 months (FU-03), and 12–15 months (FU-04) after treatment, as described previously [8]. PORPUS was completed at V01, V06, and FU-01 to FU-04. EORTC QLQ-C30 was scheduled at baseline and FU-04. IPSS and PSA were determined at baseline and during FU-01 to FU-04. Biochemical progression was defined according to national prostate cancer guidelines. In case of biochemical progression, standard imaging (CT/MRI of the abdomen and pelvis and bone scan) with the addition of PSMA PET-CT in case of negative findings was performed.

Based a literature review [8], we assumed toxicity rates of 17.5% (GU) and 10% (GI) 1 year after standard treatment. Using meta-regression, we estimated grade ≥ 2 toxicity rates for SBRT of 2.8% for the GU tract and 1.1% for the GI tract at FU-04. Sample size calculation yielded a patient number of *n* = 85 patients with a statistical power of 78% and 88% and a two-sided significance level of 5% using the exact binomial test with a Bonferroni–Holm procedure to demonstrate superiority of SBRT. A planned subgroup analysis was conducted to assess the difference between daily vs. every-other-day treatment and reported toxicity.

## Results

**Fig. 1:**
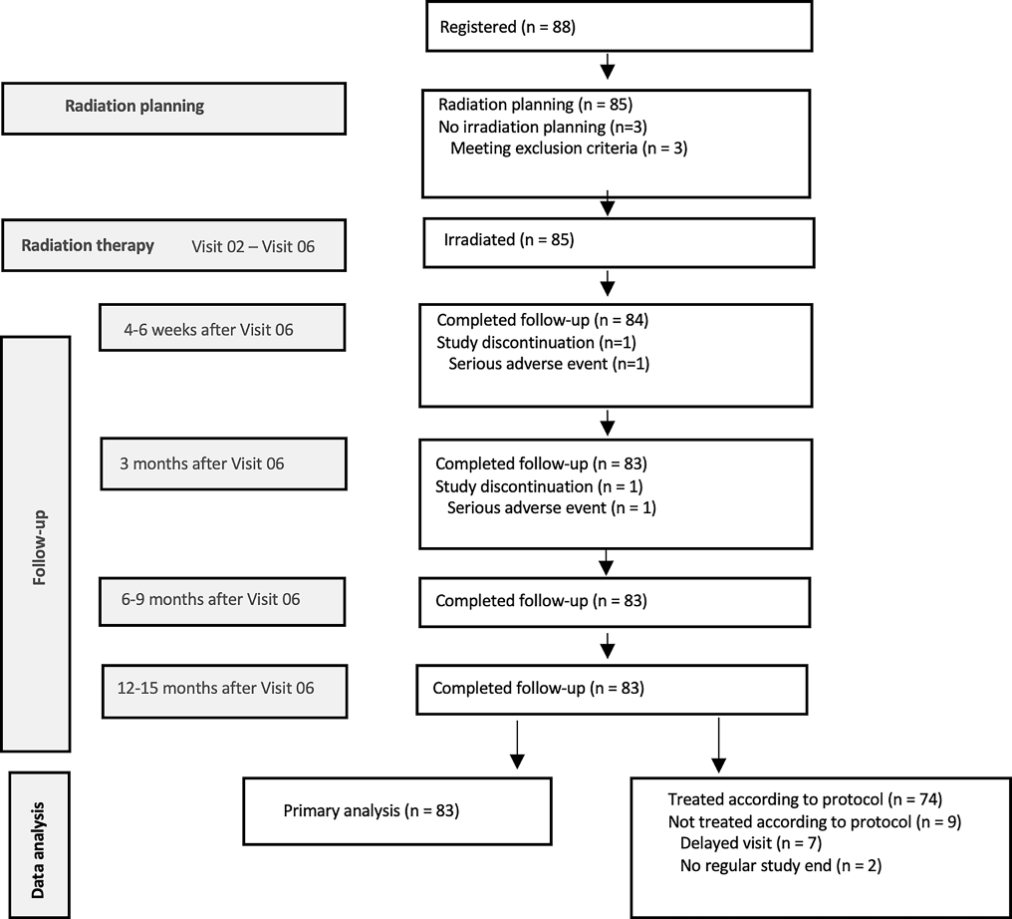
CONSORT diagram

From July 2016 through December 2018, 88 patients were enrolled. After exclusion of screening failures (*n* = 3), 85 patients received treatment at three trial sites and formed the full analysis set (FAS), which was identical to the safety analysis set in this trial. Two patients discontinued the trial due to diagnosis of pancreatic cancer (end of treatment: V06) and glioblastoma (FU-01). Thus, 83 patients were available for primary endpoint analysis. All of these patients completed the trial-mandated follow-up of 12–15 months. Per-protocol analyses (PP) were conducted using data from 74 patients with exclusion of 7 patients who had follow-up visits outside the predefined timeframe. The Consolidated Standards of Reporting Trials (CONSORT) diagram is shown in Fig. 1.

Patient characteristics are listed in Table 1. Patients mostly had intermediate-risk disease with a median PSA value of 7.97 ng/ml and a Gleason score of 7a and 7b in 43.5% and 25.9% of patients, respectively. ADT was used in only 3 patients. The dose was prescribed to the 75–83% isodose line (mean: 80%) with 92.1–98.6% coverage (mean: 95.6%), and the PTV D98% and D2% and GTV D50% were calculated at 32.6–35.2 Gy (mean: 34.0 Gy), 42.2–46.7 Gy (mean: 43.9 Gy), and 39.1–42.6 Gy (mean: 40.1 Gy), respectively. Foley catheters were not used. No major protocol dosimetry violations for critical structures were observed.

**Table 1:** Baseline characteristics of all included patients

	Median (min–max)	
*Age (years)*	72 (60–87)	
*PSA value (μg/l)*	7.97 (0.39–15.3)	
*Prostate volume (ml)*	40.0 (16–82)	
*IPSS*	5 (0–12)	
		*n* (%)
*ECOG performance score*	0	58 (68.2)
	1	27 (31.8)
*T stage*	T1a	2 (2.35)
	T1c	33 (38.82)
	T2	47 (55.29)
	T3	3 (3.53)
*Gleason score*	5	1 (1.18)
	6	25 (29.41)
	7a	37 (43.53)
	7b	22 (25.88)

*PSA* prostate-specific antigen, *IPSS* International Prostate Symptom Score, *ECOG* Eastern Cooperative Oncology Group, *min* minimum, *max* maximum

### Safety

Acute toxicity according to CTCAE-criteria was evaluated from V02 until FU-02. Overall, high-grade toxicity was rare and limited to one case each of grade 3 irritative symptoms, proctitis, and incontinence. The number of patients with the maximum grade of a toxicity according to CTCAE during the course of the study is shown in Table 2. For all adverse events reported, intensity was mostly mild, with 24 grade 2 events. The most common adverse events were irritative symptoms and proctitis.

**Table 2:** Number of patients with the maximal grade reported of each toxicity in the full analysis set (*n* = 83) according CTCAE during the study

Toxicities	Grade, *n* (%)				
	1	2	3	Missing	Total
Irritative symptoms	46 (55.42)	14 (16.87)	1 (1.20)	3 (3.61)	64 (77.11)
Obstructive symptoms	12 (14.46)	2 (2.41)	–	1 (1.20)	15 (18.07)
Nocturia	10 (12.05)	1 (1.20)	–	–	11 (13.25)
Incontinence	4 (4.82)	–	1 (1.20)	–	5 (6.02)
Hematuria	2 (2.41)	–	–	–	2 (2.41)
Proctitis	21 (25.30)	4 (4.82)	1 (1.20)	3 (3.61)	29 (34.94)
Hematochezia	1 (1.20)	–	–	–	1 (1.20)
Erectile dysfunction	5 (6.02)	2 (2.41)	–	1 (1.20)	8 (9.64)
Other	3 (3.61)	–	–	–	3 (3.61)

Late toxicity was determined using the RTOG scoring system. In the FAS, there were two cases of grade 3 bladder toxicity (2.41%), corresponding to temporary incontinence that resolved during follow-up and 5 cases of temporary grade 2 bladder toxicity (6.02%). There was no grade 2–3 GI toxicity.

**Fig. 2:**
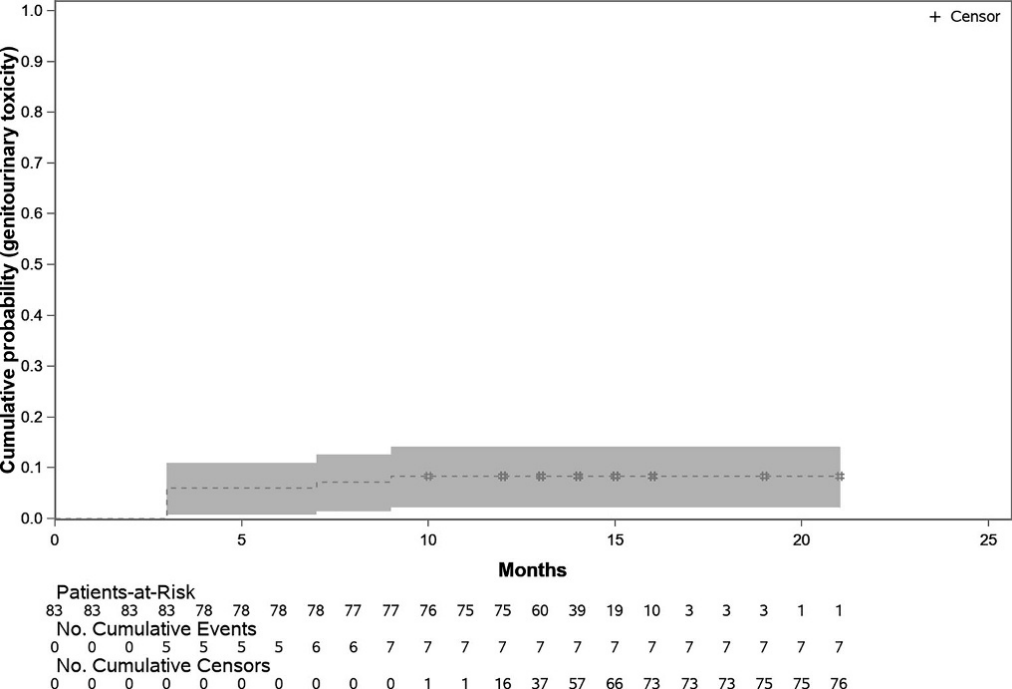
Cumulative grade ≥ 2 genitourinary toxicity in the full analysis population. Patients with a study end are censored at the respective time point. 95% confidence intervals are provided (*grey shading*)

At the final follow-up 12–15 months after treatment (FU-04), no patient suffered from treatment-related GI or GU grade ≥ 2 toxicity (Bonferroni–Holm-adjusted Wilson Score 95% confidence interval [CI] 0–5.7% for the FAS and 0–6.4% for the PP data). Thus, the null hypotheses could be rejected. The cumulative rate of grade ≥ 2 GU toxicity was 8.4% (95% CI 4.1–16.4%) in the FAS and 9.5% (95% CI 4.7–18.3%) in the PP. Figure 2 shows the cumulative grade ≥ 2 GU toxicity.

### Efficacy

**Fig. 3:**
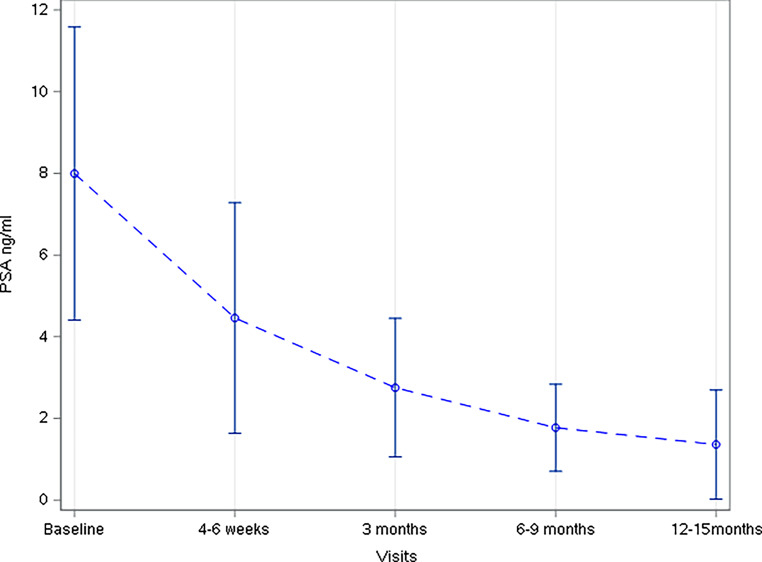
Prostate-specific antigen (PSA) kinetics during the study in the full analysis set. Mean values (*dashed line*) and standard deviations (*whiskers*) are shown

One patient had biochemical disease recurrence and developed distant metastases during follow-up. There was no local recurrence, and the median PSA value dropped to 1.18 ng/ml at the last study follow-up 12–15 months after treatment. The course of PSA measurements during the study are shown in Fig. 3.

### Quality of life and patient-reported outcomes

QOL assessment with the EORTC QLQ C30 questionnaire demonstrated no impairment of quality of life between baseline and end of study (Supplementary Table 1). Overall QOL remained stable. The median total score for FAS was 95.71 (95% CI 94.02–97.44) at baseline and 95.94 (95% CI 94.23–97.44) at FU-04.

PORPUS total scores showed little variation during the study. The median total score at baseline for FAS was 91.5 (95% CI 87.5–93.5) at baseline and 92.0 (95% CI 89.0–93.0) at FU-04. The median of difference compared to baseline was largest at V01 and FU-03 with +2 (95% CI 0–2 for V01 and 95% CI 0–4 for FU-03). The course of PORPUS total scores for FAS and PP is listed in Supplementary Table 2.

IPSS slightly increased from baseline. The median IPSS for FAS was 6.0 (95% CI 5–7) at baseline and 7.5 (95% CI 6–9) at FU-04, the median increase was 2 (95% CI 1–3). Results regarding IPSS for FAS and PP are shown in Supplementary Table 3.

**Table 3:** Late toxicity in the full analysis set (*n* = 83) according to maximum RTOG grade during the study

Toxicities	Grade, *n* (%)				*n*
	0	1	2	3	
Skin	83 (100)	–	–	–	83
Subcutaneous tissue	83 (100)	–	–	–	83
Mucous membrane	83 (100)	–	–	–	83
Salivary glands	82 (98.80)	1 (1.20)	–	–	83
Spinal cord	83 (100)	–	–	–	83
Brain	83 (100)	–	–	–	83
Eye	82 (98.80)	1 (1.20)	–	–	83
Larynx	83 (100)	–	–	–	83
Lung	82 (98.80)	1 (1.20)	–	–	83
Heart	83 (100)	–	–	–	83
Esophagus	83 (100)	–	–	–	83
Small/large intestine	81 (97.59)	2 (2.41)	–	–	83
Liver	83 (100)	–	–	–	83
Kidney	82 (98.80)	1 (1.20)	–	–	83
Bladder	73 (87.95)	3 (3.61)	5 (6.02)	2 (2.41)	83
Bone	82 (98.80)	–	1 (1.20)	–	83
Joint	83 (100)	–	–	–	83

*RTOG* Radiation Therapy Oncology Group

### Subgroup analysis of daily vs. every-other-day treatment

Results of the subgroup analysis regarding daily vs. every-other-day treatment are shown in Table 4. The majority of patients received their treatment every other day (50 patients, 60.2%). One trial center routinely used daily treatments (*n* = 33), while the other two trial centers preferentially treated patients every other day. There was a significantly higher incidence of CTCAE grade GI toxicity and proctitis as well as bladder toxicity according to the RTOG classification in the patients with daily treatment. Nocturia was more frequently documented in patients receiving their treatment every other day.

**Table 4:** Subgroup analysis according to treatment on consecutive days vs. every other day in the full analysis set

Toxicity		Consecutive, *n* (%)	Every other day, *n* (%)	Odds ratio [95% Wald CI]	*p*-value
Cumulative GI toxicity	Yes	15 (45.45)	8 (16.00)	0.23 [0.08; 0.63]	0.0054
	No	18 (54.55)	42 (84.00)		
Cumulative GU toxicity	Yes	27 (81.82)	40 (80.00)	0.89 [0.29; 2.73]	1
	No	6 (18.18)	10 (20.00)		
Incontinence	Yes	2 (6.06)	3 (6.00)	0.99 [0.16; 6.27]	1
	No	31 (93.94)	47 (94.00)		
Nycturia	Yes	1 (3.03)	10 (20.00)	8.00 [0.97; 65.82]	0.0434
	No	32 (96.97)	40 (80.00)		
Proctitis	Yes	21 (63.64)	8 (16.00)	0.11 [0.04; 0.31]	0.00001
	No	12 (36.36)	42 (84.00)		
Erectile dysfunction	Yes	5 (15.15)	3 (6.00)	0.36 [0.08; 1.61]	0.2553
	No	28 (84.85)	47 (94.00)		
Irritative complaints	Yes	27 (81.82)	38 (76.00)	0.70 [0.23; 2.11]	0.5957
	No	6 (18.18)	12 (24.00)		
Obstructive complaints	Yes	4 (12.12)	11 (22.00)	2.04 [0.59; 7.07]	0.3830
	No	29 (87.88)	39 (78.00)		
Bladder (RTOG)	Yes	7 (21.21)	0 (0.00)	0.04 [0.002; 0.64]	0.0010
	No	26 (78.79)	50 (100.00)		

*GI* gastrointestinal, *GU* genitourinary, *RTOG* Radiation Therapy Oncology Group, *CI* confidence interval

## Discussion

Our results demonstrate the feasibility and tolerability of robotic SBRT with 35 Gy in 5 fractions for patients with localized prostate cancer.

Since conception of the HYPOSTAT trial, evidence for SBRT in the treatment of patients with prostate cancer has considerably improved. In 2019, a meta-analysis of 38 prospective single-arm clinical trials, case series, and registries with a total of 6116 patients was published [10]. Biochemical control was high, with a 5-year rate of 95.3% (95% CI, 91.3–97.5%). Acute GU and GI grade 2 toxicity occurred in 15.5% and 6.1%, respectively. Late grade 2 toxicity occurred in 12.1% and 4.9% of patients for the GU and GI tract, respectively. An increasing biologically effective dose was associated with increased biochemical control, but also with increased late grade ≥ 3 GU toxicity [10].

To date, two randomized controlled trials have reported results. The HYPO-RT-PC-trial randomized 1200 patients to definitive radiotherapy with 78 Gy in 39 fractions or to SBRT with 42.7 Gy in 7 fractions [11]. In this trial, 80% of patients were treated with 3D conformal radiotherapy and 90% of patients had fiducials implanted. Almost 90% of patients had intermediate-risk disease, as patients with low-risk disease could not be enrolled. Non-inferiority of SBRT in terms of failure-free survival was demonstrated. There was a slight but significant increase in acute GU and GI toxicity up to 1 year in the case of GU toxicity, but long-term toxicity was similar between the two arms. The PACE‑B trial compared SBRT with 5 × 7.25 Gy to conventional or moderate hypofractionation in a trial population of 874 patients with low- or intermediate-risk prostate cancer [12]. SBRT was delivered with robotic SBRT in 41% of patients and with conventional linear accelerators in 59% of patients. Only 73% of patients in the SBRT arm received fiducials. All patients in the standard arm received intensity-modulated radiotherapy. Acute toxicity results up to 12 weeks after the end of radiotherapy have been published [12]. Worst acute toxicity grade ≥ 2 according to RTOG was similar between the arms. Toxicity grading according to CTCAE showed significantly higher rates of grade ≥ 2 GU and GI acute toxicity with SBRT. Toxicity outcomes at 2 years were published recently [13]. Late grade ≥ 2 GU toxicity at 2 years according to RTOG criteria was similar between the treatment arms (2% vs. 3%); however, when studying CTCAE-documented toxicity, patients treated with SBRT had a higher incidence of grade ≥ 2 GU toxicity (+5.7%; *p* < 0.01). Similarly, cumulative GU toxicity according to RTOG and CTCAE was higher in the SBRT arm. Late GI grade ≥ 2 toxicity was comparable between the treatment groups.

The optimal treatment technique, image guidance, and dose prescription for SBRT in prostate cancer are unknown. While SBRT may be delivered using standard linear accelerators with CT-based image guidance, there are some data to suggest that treatment techniques with intrafractional image guidance and advanced motion management may improve outcome. A subgroup analysis from the PACE‑B trial according to treatment technique in the SBRT arm demonstrated significantly lower rates of worst RTOG grade ≥ 2 acute GU toxicity for patients treated with robotic SBRT as compared to conventional linear accelerators, while GI toxicity was similar [12]. Subgroup analysis at 2 years showed significantly lower rates of late grade ≥ 2 GU and GI toxicity with robotic SBRT compared to C‑arm-based SBRT; however, this was a non-randomized comparison and confounding factors may have influenced the results [13]. Nevertheless, these results regarding reduced GU toxicity may be attributable to a reduced dose to the bladder neck because of the use of multiple non-coplanar beam angles with robotic SBRT. Another explanation would be the use of reduced PTV margins; however, GI toxicity was similar in the PACE‑B trial.

In the past years, MR-guided radiotherapy has been introduced into clinical use, which offers the benefit of continuous intrafractional image guidance and the possibility of gated dose delivery [14]. Recently, a planned interim analysis from the randomized controlled MIRAGE trial was presented at the American Society of Clinical Oncology (ASCO) GU meeting 2022 [15]. All patients received SBRT with 40 Gy in 5 fractions but were randomized either to standard treatment with CT-based image guidance or to MR-guided radiotherapy. Of note, a PTV margin of 2 mm was used with MR-guided radiotherapy, while 4 mm was applied for CT-guided radiotherapy. Acute grade ≥ 2 GU and GI toxicity was significantly reduced with MR-guided radiotherapy. No results regarding late toxicity or efficacy are available as of now. These data suggest that advanced treatment techniques may result in improvements regarding acute and late toxicity. Further research is needed and prospective trials regarding MR-guided radiotherapy in prostate cancer are ongoing [16].

In prostate cancer, a clear relationship between radiotherapy dose and biochemical control has been demonstrated. This has also been established for SBRT [10]. Our trial used a dose of 35 Gy in 5 fractions, which is at the lower end of the dose spectrum for prostate SBRT. Due to differences in dose homogeneity inside the prostate between C‑arm-based and robotic SBRT, a direct comparison between treatment schedules and trial results is somewhat limited. Our study showed a median PSA of 1.18 ng/ml at the last follow-up at 12–15 months, which is comparable to multi-institutional analysis of PSA kinetics after SBRT [17]. Since the PSA nadir was reached 40 months after radiotherapy in this analysis, no definitive conclusions can be drawn from our data at this point. Nevertheless, prospective data from a cohort of 230 patients with low-risk prostate cancer showed a comparable outcome with 35 Gy compared to 36.25 Gy in 5 fractions at 10 years [18].

The preplanned subgroup analysis of treatment frequency demonstrated a higher incidence of acute GI toxicity and late GU toxicity in patients who had daily treatment. This analysis should be regarded as hypothesis generating, as treatment patterns were mostly site associated. In the PACE‑B trial, individual documentation as well as thresholds for prescribing α‑antagonists triggering GU adverse event severity have been shown to considerably differ between physicians and centers [13], which may partly explain our findings. This also concerns the isolated finding of increased nocturia in the every-other-day group, which is in contrast to the increased toxicity seen for other GU and GI toxicity items. Nevertheless, data from the randomized phase II PATRIOT trial showed that a more protracted schedule with weekly treatments as compared to every-other-day treatment significantly reduced acute GI and GU toxicity, highlighting the role of overall treatment time [19, 20].

Limitations of this report are the short overall follow-up and the associated lack of definitive oncological outcome data. Nevertheless, long-term data for 35 Gy in 5 fractions have been published previously. Due to the limited inclusion criteria, we cannot make any statement regarding the use of robotic SBRT for patients with high-risk prostate cancer. The optimal combination of SBRT and ADT remains to be established. Toxicity assessment according to RTOG criteria seems to have a limited sensitivity for detecting moderate GU toxicity, as suggested by the recently reported results of the PACE‑B trial [13]. The results of the subgroup analysis should be interpreted with caution. Furthermore, regarding the analysis of acute toxicities according to CTCAE, the absence of a toxicity was not explicitly reported. The strengths of this analysis include the rigorous quality assurance and collection of patient-reported outcomes and quality of life data.

In summary, our results demonstrate short-term feasibility and tolerability of robotic SBRT for patients with localized prostate cancer. Further research and longer follow-up are necessary to validate this dose regimen and to analyze the role of treatment technique and dose prescription as well as escalation in prostate SBRT.

## Supplementary Information

Supplementary Table 1: Results of quality of life measured with the EORTC QLQ-C30 questionnaire.

Supplementary Table 2a: Results of PORPUS total score—comparison to visit 0 (baseline). Supplementary Table 2b: Results of PORPUS for each domain and total presented as mean and standard deviation.

Supplementary Table 3a: International Prostate Symptom Score (IPSS). Supplementary Table 3b: International Prostate Symptom Score (IPSS)—comparison to screening.

Supplementary Fig. 1: PSA kinetics during the study in the full analysis set. Each line represents one individual patient.
