# Supplementary material for: Robotic stereotactic body radiotherapy for localized prostate cancer: final analysis of the German HYPOSTAT trial
Source: Strahlenther Onkol. 2023 Feb 9;199(6):565–73. doi: 10.1007/s00066-023-02044-2 (PMC10212861; doi:10.1007/s00066-023-02044-2)
Supplement: Supplementary file 1 — Supplementary Table 1: Results of quality of life measured with the EORTC QLQ-C30 questionnaire. [file 66_2023_2044_MOESM1_ESM.docx]

Supplementary Table 1: Results of quality of life measured with the EORTC QLQ-C30 questionnaire.

| **Analysis set** | **Scale** | **Before irradation (Visit 0)** | | | | **After irradiation (end of study, FU 4)** | | | | **Before vs. after** |
| --- | --- | --- | --- | --- | --- | --- | --- | --- | --- | --- |
|  |  | **Min** | **Max** | **IQR** | **Median**  **[95%-CI]** | **Min** | **Max** | **IQR** | **Median**  **[95%-CI]** | **Median of difference**  **[95%-CI]** |
| **FAS** | **QL2** | 33.33 | 100 | 8.33 | 83.33  [83.33 - 83.33] | 16.67 | 100 | 25.00 | 83.33  [83.33 - 83.33] | 0.00  [0.00 -0.00] |
|  | **PF2** | 46.67 | 100 | 6.67 | 100  [100 - 100] | 46.67 | 100 | 6.67 | 100  [93.33 – 100] | 0.00  [0.00 -0.00 |
|  | **RF2** | 33.33 | 100 | 0.00 | 100  [100 - 100] | 46.67 | 100 | 0.00 | 100  [100 - 100] | 0.00  [0.00 -0.00 |
|  | **EF** | 41.67 | 100 | 25.00 | 83.33  [83.33 – 100] | 25.00 | 100 | 16.67 | 100  [91.68 – 100] | 0.00  [0.00 -8.33] |
|  | **CF** | 33.33 | 100 | 16.67 | 100  [100 - 100] | 46.67 | 100 | 16.67 | 100  [83.33 – 100] | 0.00  [0.00 -0.00 |
|  | **SF** | 33.33 | 100 | 16.67 | 100  [100 - 100] | 46.67 | 100 | 0.00 | 100  [100 - 100] | 0.00  [0.00 -0.00 |
|  | **FA** | 0.00 | 77.78 | 11.11 | 0.00  [0.00 – 11.11] | 0.00 | 88.89 | 22.22 | 0.00  [0.00 – 11.11] | 0.00  [0.00 -0.00 |
|  | **NV** | 0.00 | 16.67 | 0.00 | 0.00  [0.00 – 0.00] | 0.00 | 16.67 | 0.00 | 0.00  [0.00 – 0.00] | 0.00  [0.00 -0.00 |
|  | **PA** | 0.00 | 83.33 | 8.33 | 0.00  [0.00 – 0.00] | 0.00 | 66.67 | 0.00 | 0.00  [0.00 – 0.00] | 0.00  [0.00 -0.00] |
|  | **DY** | 0.00 | 66.67 | 0.00 | 0.00  [0.00 – 0.00] | 0.00 | 66.67 | 0.00 | 0.00  [0.00 – 0.00] | 0.00  [0.00 -0.00] |
|  | **SL** | 0.00 | 66.67 | 33.33 | 0.00  [0.00 – 0.00] | 0.00 | 66.67 | 33.33 | 0.00  [0.00 – 0.00] | 0.00  [0.00 -0.00] |
|  | **AP** | 0.00 | 66.67 | 0.00 | 0.00  [0.00 – 0.00] | 0.00 | 66.67 | 0.00 | 0.00  [0.00 – 0.00] | 0.00  [0.00 -0.00] |
|  | **CO** | 0.00 | 66.67 | 0.00 | 0.00  [0.00 – 0.00] | 0.00 | 66.67 | 0.00 | 0.00  [0.00 – 0.00] | 0.00  [0.00 -0.00] |
|  | **DI** | 0.00 | 100 | 0.00 | 0.00  [0.00 – 0.00] | 0.00 | 66.67 | 0.00 | 0.00  [0.00 – 0.00] | 0.00  [0.00 -0.00] |
|  | **FI** | 0.00 | 66.67 | 0.00 | 0.00  [0.00 – 0.00]] | 0.00 | 66.67 | 0.00 | 0.00  [0.00 – 0.00] | 0.00  [0.00 -0.00] |
|  | **Total** | 51.45 | 100 | 6.73 | 95.71  [94.02– 97.44] | 51.88 | 100 | 11.20 | 95.94  [94.23 – 97.44] | 0.00  [-0.51 – 0.64] |
| **PP** | **QL2** | 33.33 | 100 | 8.33 | 83.33  [83.33 - 83.33] | 16.67 | 100 | 16.67 | 83.33  [83.33 - 83.33 | 0.00  [0.00 -0.00] |
|  | **PF2** | 46.67 | 100 | 6.67 | 100  [100 - 100] | 46.67 | 100 | 6.67 | 100  [93.33 – 100] | 0.00  [0.00 -0.00] |
|  | **RF2** | 33.33 | 100 | 0.00 | 100  [100 - 100] | 33.33 | 100 | 0.00 | 100  [100 - 100] | 0.00  [0.00 -0.00] |
|  | **EF** | 41.67 | 100 | 25.00 | 83.33  [75.00 – 1.68] | 33.33 | 100 | 16.67 | 100  [91.67 – 100] | 0.00  [0.00 -8.33] |
|  | **CF** | 33.33 | 100 | 0.00 | 100  [100 - 100] | 33.33 | 100 | 16.67 | 100  [83.33 – 100] | 0.00  [0.00 -0.00] |
|  | **SF** | 33.33 | 100 | 16.67 | 100  [100 - 100] | 33.33 | 100 | 0.00 | 100  [100 - 100] | 0.00  [0.00 -0.00] |
|  | **FA** | 0.00 | 77.78 | 11.11 | 0.00  [0.00 – 11.11] | 0.00 | 61.11 | 22.22 | 0.00  [0.00 -16.67] | 0.00  [0.00 -0.00] |
|  | **NV** | 0.00 | 16.67 | 0.00 | 0.00  [0.00 – 0.00 | 0.00 | 16.67 | 0.00 | 0.00  [0.00 – 0.00 | 0.00  [0.00 -0.00] |
|  | **PA** | 0.00 | 83.33 | 16.67 | 0.00  [0.00 – 0.00 | 0.00 | 66.67 | 0.00 | 0.00  [0.00 – 0.00 | 0.00  [0.00 -0.00] |
|  | **DY** | 0.00 | 66.67 | 0.00 | 0.00  [0.00 – 0.00 | 0.00 | 66.67 | 0.00 | 0.00  [0.00 – 0.00 | 0.00  [0.00 -0.00] |
|  | **SL** | 0.00 | 66.67 | 33.33 | 0.00  [0.00 – 0.00 | 0.00 | 66.67 | 33.33 | 0.00  [0.00 – 0.00 | 0.00  [0.00 -0.00] |
|  | **AP** | 0.00 | 66.67 | 0.00 | 0.00  [0.00 – 0.00 | 0.00 | 66.67 | 0.00 | 0.00  [0.00 – 0.00 | 0.00  [0.00 -0.00] |
|  | **CO** | 0.00 | 66.67 | 0.00 | 0.00  [0.00 – 0.00 | 0.00 | 66.67 | 0.00 | 0.00  [0.00 – 0.00 | 0.00  [0.00 -0.00] |
|  | **DI** | 0.00 | 100 | 0.00 | 0.00  [0.00 – 0.00 | 0.00 | 66.67 | 0.00 | 0.00  [0.00 – 0.00 | 0.00  [0.00 -0.00] |
|  | **FI** | 0.00 | 66.67 | 0.00 | 0.00  [0.00 – 0.00 | 0.00 | 66.67 | 0.00 | 0.00  [0.00 – 0.00 | 0.00  [0.00 -0.00] |
|  | **Total** | 51.45 | 100 | 6.62 | 95.41  [93.50 –96.92] | 51.88 | 100 | 10.56 | 95.73  [93.93 – 96.92] | 0.00  [-0.64 - 0.64] |
| For median and for median difference, 95% distribution-free confidence intervals (CI) were calculated. QL2 = global health status; PF2 = physical functioning; RF2 = role functioning; EF = emotional functioning; CF = cognitive functioning; SF = sexual functioning; FA = fatigue; NV = nausea and vomiting; PA = pain; DY = Dyspnea; SL = Insomnia; AP = appetite loss; CO = constipation; DI = diarrhea; FI = financial difficulties. | | | | | | | | | | |
