# Supplementary material for: Robotic stereotactic body radiotherapy for localized prostate cancer: final analysis of the German HYPOSTAT trial
Source: Strahlenther Onkol. 2023 Feb 9;199(6):565–73. doi: 10.1007/s00066-023-02044-2 (PMC10212861; doi:10.1007/s00066-023-02044-2)
Supplement: Supplementary file 2 — Supplementary Table 2a: Results of PORPUS total score—comparison to visit 0 (baseline). Supplementary Table 2b: Results of PORPUS for each domain and total presented as mean and standard deviation. [file 66_2023_2044_MOESM2_ESM.docx]

Supplementary Table 2a: Results of PORPUS total score – comparison to visit 0 (baseline)

| **Analysis set** | **Time point** | **n*** | **Min** | **Max** | **IQR** | **Median**  **[95%-CI]** | **Median of difference**  **[95%-CI]** |
| --- | --- | --- | --- | --- | --- | --- | --- |
| **FAS** | Visit 0 | 81 | 63.00 | 100 | 9.83 | 91.50  [87.50 – 93-50] | - |
|  | Visit 1 | 51 | 77.00 | 100 | 9.00 | 93.50  [91.83 – 96.00] | 2.00  [0.00 – 2.00] |
|  | Visit 6 | 83 | 58.50 | 100 | 10.17 | 89.50  [87.50 – 90.33] | -0.43  [-3.00 – 0.50] |
|  | FU 1 | 79 | 65.83 | 100 | 10.00 | 90.00  [87.50 – 92.00] | 0.00  [-2.50 – 1.25]] |
|  | FU 2 | 75 | 64.58 | 100 | 10.67 | 92.00  [89.50 – 94.00] | 1.79  [0 – 2.50] |
|  | FU 3 | 75 | 72.50 | 100 | 12.00 | 90.42  [88.33 – 94.00] | 2.00  [0 – 4.00] |
|  | FU 4 | 81 | 59.50 | 100 | 12.00 | 92.00  [89.00 – 93.00] | 0.00  [-1.50 – 1.67] |
| **PP** | Visit 0 | 70 | 63.00 | 100 | 10.00 | 91.50  [87.50 – 93.50] | - |
|  | Visit 1 | 43 | 77.00 | 100 | 11.50 | 92.50  [89.50 – 95.56] | 2.00  [0.00 – 2.63] |
|  | Visit 6 | 73 | 58.50 | 100 | 9.67 | 89.00  [86.33 – 90.00] | -0.50  [-3.67 – 0.50] |
|  | FU 01 | 70 | 65.83 | 100 | 9.50 | 89.50  [86.50 – 91.83] | 0.00  [-2.50 – 1.00]] |
|  | FU 02 | 67 | 64.58 | 100 | 12.00 | 90.83  [89.50 – 94.00] | 2.00  [0 – 3.13] |
|  | FU 03 | 68 | 72.50 | 100 | 12.17 | 90.00  [88.00 – 93.50] | 2.50  [0.50 – 4.00] |
|  | FU 04 | 72 | 65.00 | 100 | 11.92 | 90.58  [88.00 – 92.67] | 0.00  [-1.50 – 1.56] |
| *Number of non-missing values; min = minimum; max = maximum; IQR = inter-quartile range; 95% CI = 95%-confidence interval; FAS = full analysis set, PP = per protocol; FU = follow-up (FU 1 = 4-6 weeks after last day of irradiation; FU 2 = 2 months +/- 1 week after last day of irradiation; FU 3 = 6-9 months after last day of irradiation and FU 4 = 12-15 months after last day of irradiation). For median and for median difference, 95% distribution-free CIs were calculated. | | | | | | | |

Supplementary Table 2b: Results of PORPUS for each domain and total presented as mean and standard deviation

|  | | **PORPUS domain** | | | | | | | | | |  |
| --- | --- | --- | --- | --- | --- | --- | --- | --- | --- | --- | --- | --- |
| **Analysis set** | **Time point** | **Pain and disturbing body sensations** | **Energy** | **Support from family and friends** | **Communication with doctor** | **Emotional well-being** | **Urinary frequency** | **Leaking and poor bladder control** | **Sexual function** | **Sexual drive and interest** | **Bowel symptoms** | **Total** |
| **FAS** | Visit 0 | 1.55 (0.80) | 2.21 (0.66) | 1.20 (0.55) | 1.16 (0.39) | 1.35 (0.53) | 1.61 (0.60) | 1.22 (0.54) | 2.01 (1.15) | 1.89 (1.03) | 1.13 (0.38) | 88.93 (7.83) |
|  | Visit 1 | 1.21 (0.57) | 1.96 (0.71) | 1.13 (0.34) | 1.10 (0.36) | 1.23 (0.47) | 1.42 (0.54) | 1.08 (0.27) | 1.96 (1.22) | 1.78 (1.03) | 1.14 (0.35) | 92.05 (6.74) |
|  | Visit 6 | 1.58 (0.96) | 2.16 (0.81) | 1.15 (0.47) | 1.05 (0.22) | 1.41 (0.60) | 1.99 (0.72) | 1.32 (0.60) | 2.29 (1.31) | 2.02 (1.10) | 1.39 (0.62) | 87.03 (9.17) |
|  | FU 01 | 1.26 (0.63) | 2.07 (0.81) | 1.19 (0.50) | 1.05 (0.22) | 1.25 (0.46) | 1.89 (0.66) | 1.41 (0.73) | 2.13 (1.19) | 1.87 (1.10) | 1.35 (0.50) | 88.73 (7.36) |
|  | FU 02 | 1.35 (0.77) | 2.03 (0.78) | 1.14 (0.41) | 1.07 (0.25) | 1.30 (0.54) | 1.62 (0.61) | 1.29 (0.58) | 2.10 (1.15) | 1.88 (1.12) | 1.23 (0.54) | 89.87 (8.30) |
|  | FU 03 | 1.27 (0.66) | 2.12 (0.85) | 1.08 (0.32) | 1.07 (0.25) | 1.26 (0.53) | 1.68 (0.72) | 1.30 (0.57) | 2.03 (1.09) | 1.89 (1.14) | 1.18 (0.39) | 90.21 (7.40) |
|  | FU 04 | 1.41 (0.76) | 2.07 (0.74) | 1.17 (0.52) | 1.07 (0.26) | 1.27 (0.50) | 1.80 (0.71) | 1.37 (0.68) | 2.25 (1.20) | 1.93 (1.15) | 1.23 (0.45) | 88.61 (8.85) |
| **PP** | Visit 0 | 1.56 (0.84) | 2.23 (0.66) | 1.16 (0.47) | 1.15 (0.39) | 1.38 (0.54) | 1.63 (0.62) | 1.19 (0.52) | 2.04 (1.18) | 1.96 (1.20) | 1.14 (0.39) | 88.67 (7.96) |
|  | Visit 1 | 1.24 (0.61) | 2.04 (0.67) | 1.14 (0.35) | 1.11 (0.39) | 1.25 (0.49) | 1.45 (0.55) | 1.07 (0.25) | 2.10 (1.27) | 1.90 (1.05) | 1.17 (0.38) | 91.08 (6.78) |
|  | Visit 6 | 1.60 (0.99) | 2.23 (0.80) | 1.14 (0.48) | 1.05 (0.21) | 1.44 (0.62) | 2.01 (0.71) | 1.30 (0.59) | 2.38 (136) | 2.11 (1.12) | 1.42 (0.64) | 86.34 (9.41) |
|  | FU 01 | 1.28 (0.65) | 2.10 (0.79) | 1.19 (0.52) | 1.06 (0.23) | 1.26 (0.47) | 1.89 (0.68) | 1.41 (0.73) | 2.20 (1.23) | 1.93 (1.14) | 1.38 (0.51) | 88.14 (7.33) |
|  | FU 02 | 1.39 (0.81) | 2.03 (0.77) | 1.15 (0.43) | 1.07 (0.26) | 1.32 (0.56) | 1.65 (0.62) | 1.29 (0.59) | 2.17 (1.18) | 1.91 (1.09) | 1.25 (0.55) | 89.93 (8.50) |
|  | FU 03 | 1.29 (0.69) | 2.12 (0.80) | 1.09 (0.33) | 1.06 (0.24) | 1.26 (0.50) | 1.70 (0.74) | 1.30 (0.58) | 2.09 (1.11) | 1.92 (1.10) | 1.20 (0.40) | 89.91 (7.35) |
|  | FU 04 | 1.40 (0.76) | 2.08 (0.71) | 1.19 (0.54) | 1.07 (0.25) | 1.27 (0.48) | 1.79 (0.68) | 1.38 (0.64) | 2.30 (1.22) | 1.99 (1.16) | 1.26 (0.47) | 88.30 (8.38) |
| FAS = full analysis set; PP = per protocol; FU = follow-up (FU 01 = 4-6 weeks after last day of irradiation; FU 02 = 2 months +/- 1 week after last day of irradiation; FU 03 = 6-9 months after last day of irradiation and FU 04 = 12-15 months after last day of irradiation). | | | | | | | | | | | | |
