# Supplementary material for: Robotic stereotactic body radiotherapy for localized prostate cancer: final analysis of the German HYPOSTAT trial
Source: Strahlenther Onkol. 2023 Feb 9;199(6):565–73. doi: 10.1007/s00066-023-02044-2 (PMC10212861; doi:10.1007/s00066-023-02044-2)
Supplement: Supplementary file 3 — Supplementary Table 3a: International Prostate Symptom Score (IPSS). Supplementary Table 3b: International Prostate Symptom Score (IPSS)—comparison to screening. [file 66_2023_2044_MOESM3_ESM.docx]

Supplementary Table 3a: International prostate symptom score (IPSS)

| **Analysis set** | **Time point** | **Raw** | **Difference to screening** |
| --- | --- | --- | --- |
|  |  | **Mean (SD)** | **Mean (SD)** |
|  |  | 6.43 (3.69) | - |
| **FAS** | Screening |  |  |
|  | FU 01 | 8.95 (6.28) | 2.59 (5.79) |
|  | FU 02 | 7.43 /6.06) | 1.09 (5.56) |
|  | FU 03 | 8.02 (6.47) | 1.62 (6.22) |
|  | FU 04 | 8.95 (6.28) | 2.59 (5.79) |
| **PP** | Screening | 6.56 (3.75) | - |
|  | FU 01 | 9.14 (6.31) | 2.55 (5.77) |
|  | FU 02 | 8.01 (6.16) | 1.44 (5.68) |
|  | FU 03 | 8.54 (6.55) | 1.89 (6.45) |
|  | FU 04 | 9.14 (6.31) | 2.55 (5.77) |
| FAS = full analysis set; PP = per protocol; FU = follow-up (FU 01 = 4-6 weeks after last day of irradiation; FU 02 = 2 months +/- 1 week after last day of irradiation; FU 03 = 6-9 months after last day of irradiation and FU 04 = 12-15 months after last day of irradiation); SD = standard deviation. | | | |

Supplementary Table 3b: International prostate symptom score (IPSS) – comparison to screening

| **Analysis set** | **Time point** | **n*** | **Min** | **Max** | **IQR** | **Median**  **[95% CI]** | **Median of difference**  **[95% CI]** |
| --- | --- | --- | --- | --- | --- | --- | --- |
| **FAS** | Screening | 85 | 0.00 | 14.00 | 6.00 | 6.00  [5.00 – 7.00] | - |
|  | FU 01 | 82 | 1.00 | 32.50 | 7.00 | 7.50  [6.00 – 9.00] | 2.00  [1.00 – 3.00] |
|  | FU 02 | 81 | 0.00 | 27.00 | 8.00 | 6.00  [5.00 – 8.00] | 0.00  [-1.00 – 2.00] |
|  | FU 03 | 78 | 0.00 | 34.00 | 8.00 | 7.00  [5.00 – 8.00] | 0.00  [-1.00 – 1.00] |
|  | FU 04 | 82 | 1.00 | 32.50 | 7.00 | 7.50  [6.00 – 9.00] | 2.00  [1.00 - 3.00] |
| **PP** | Screening | 74 | 0.00 | 14.00 | 7.00 | 6.00  [5.00 – 7.00] | - |
|  | FU 01 | 73 | 1.00 | 32.50 | 7.00 | 8.00  [6.00 – 9.00] | 2.00  [1.00 – 3.00] |
|  | FU 02 | 72 | 0.00 | 27.00 | 7.00 | 7.00  [5.00 – 9.00] | 0.50  [-1.00 – 2.00] |
|  | FU 03 | 70 | 0.00 | 34.00 | 8.50 | 7.00  [6.00 – 9.00] | 0.00  [-1.00 – 1.50] |
|  | FU 04 | 73 | 1.00 | 32.50 | 7.00 | 8.00  [6.00 – 9.00] | 2.00  [1.00 - 3.00 |
| *Number of non-missing values; min = minimum; max = maximum; IQR = inter-quartile range; 95% CI = 95%-confidence interval; FAS = full analysis set, PP = per protocol; FU = follow-up (FU 01 = 4-6 weeks after last day of irradiation; FU 02 = 2 months +/- 1 week after last day of irradiation; FU 03 = 6-9 months after last day of irradiation and FU 04 = 12-15 months after last day of irradiation). For median and for median difference, 95% distribution-free CIs were calculated. | | | | | | | |
