# Supplementary figures and images for: Robotic stereotactic body radiotherapy for localized prostate cancer: final analysis of the German HYPOSTAT trial
Source: Strahlenther Onkol. 2023 Feb 9;199(6):565–73. doi: 10.1007/s00066-023-02044-2 (PMC10212861; doi:10.1007/s00066-023-02044-2)

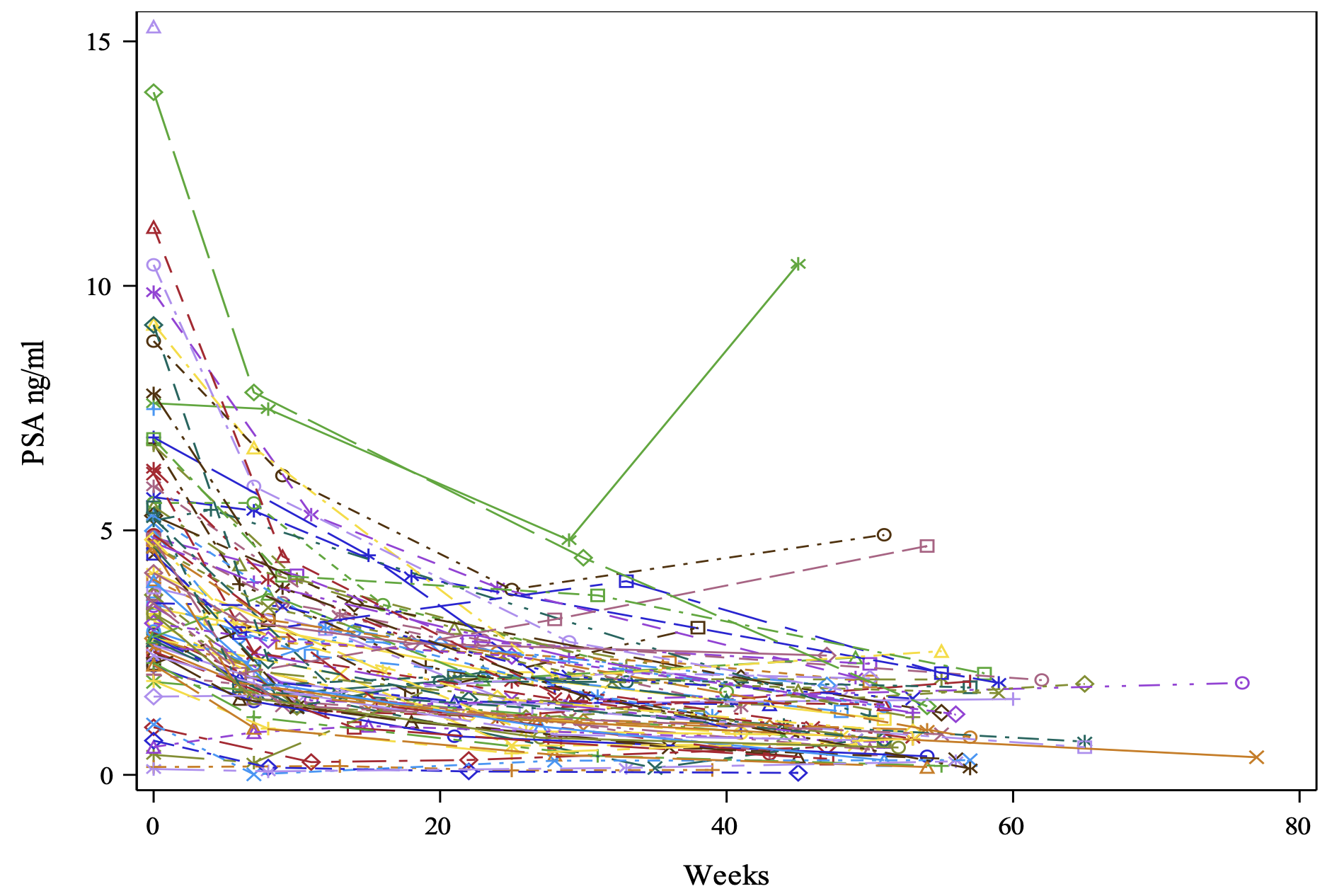

Supplement: Supplementary file 4 — Supplementary Fig. 1: PSA kinetics during the study in the full analysis set. Each line represents one individual patient. [file 66_2023_2044_MOESM4_ESM.png]
